# Trends in incidence and in short term survival following a subarachnoid haemorrhage in Scotland, 1986 - 2005: a retrospective cohort study

**DOI:** 10.1186/1471-2377-11-38

**Published:** 2011-03-29

**Authors:** Karen J Macpherson, James D Lewsey, Pardeep S Jhund, Michelle Gillies, Jim WT Chalmers, Adam Redpath, Andrew Briggs, Matthew Walters, Peter Langhorne, Simon Capewell, John JV McMurray, Kate MacIntyre

**Affiliations:** 1 NHS Quality Improvement Scotland, Glasgow G1 2NP, UK; 2 Department of Public Health, University of Glasgow, Glasgow, UK; 3 British Heart Foundation, Cardiovascular Research Centre, University of Glasgow, Glasgow, UK; 4 ISD, Edinburgh, UK; 5 Cardiovascular and Medical Sciences, University of Glasgow, Glasgow, UK; 6 Department of Public Health, University of Liverpool, UK

## Abstract

**Background:**

To examine age and sex specific incidence and 30 day case fatality for subarachnoid haemorrhage (SAH) in Scotland over a 20 year period.

**Methods:**

A retrospective cohort study using routine hospital discharge data linked to death records.

**Results:**

Between 1986 and 2005, 12,056 individuals experienced an incident SAH. Of these 10,113 (84%) survived to reach hospital. Overall age-standardised incidence rates were greater in women than men and remained relatively stable over the study period. In 2005, incidence in women was 12.8 (95% CI 11.5 to 14.2) and in men 7.9 (95% CI 6.9 to 9.1). 30 day case fatality in individuals hospitalised with SAH declined substantially, falling from 30.0% in men and 33.9% in women in 1986-1990 to 24.5% in men and 29.1% in women in 2001-2005. For both men and women, the largest reductions were observed in those aged between 40 to 59 years. After adjustment for age, socio-economic status and co-morbidity, the odds of death at 30 days in 2005 compared to odds of death in 1986 was 0.64 (0.54 to 0.76), p < 0.001 for those below 70 years, and 1.14 (0.83 to 1.56), p = 0.4 in those 70 years and above.

**Conclusions:**

Incidence rates for SAH remained stable between 1986 and 2005 suggesting that a better understanding of SAH risk factors and their reduction is needed. 30 day case fatality rates have declined substantially, particularly in middle-age. However, they remain high and it is important to ensure that this is not due to under-diagnosis or under-treatment.

## Background

Although subarachnoid haemorrhage (SAH) accounts for only 5% of all strokes[1], its impact on the loss of productive life years is similar to that of ischaemic stroke[2]. Nearly one third of all stroke related years of potential life lost before age 65 are attributable to SAH[3]. Recognised risk factors for SAH include hypertension, alcohol excess and smoking[4].

An understanding of the epidemiology of SAH over time enables strategic planning of treatment services, ensures the appropriate targeting of preventive measures and allows assessment of the effectiveness of existing management strategies. Effective therapies include oral nimodipine[5], neurosurgical clipping and endo-vascular coiling[6].

Existing studies have largely been confined to specific age groups, do not present data separately for men and women and have not reported detailed co-morbidity or socioeconomic status. Furthermore, description of secular trends in incidence and case fatality are limited and inconsistent[7-10].

The aim of the present study is to provide a comprehensive description of temporal trends in incidence and 30 day case fatality of SAH in Scotland using routinely collected hospital discharge data and death record data for the entire country over the period 1986 to 2005.

## Methods

All emergency hospital care is provided by the National Health Service in Scotland (NHSS). Details of all NHSS hospital discharges are recorded centrally and these are linked at an individual patient level to subsequent death anywhere in the United Kingdom. Discharge data recorded include up to six diagnoses (one principal and five secondary), coded according to the World Health Organisation Classification of Diseases, age, sex, postcode of residence, date of hospitalisation, length of stay and specialty.

A first hospitalisation for subarachnoid haemorrhage was defined as one with a principal diagnosis of subarachnoid haemorrhage (International Classification of Diseases 9th Revision 430 and International Classification of Diseases 10th Revision I60) and no previous hospitalisation (principal or secondary position) for cerebrovascular disease (ICD9 430-434, ICD10 I60 to I69) within the previous five years. Traumatic subarachnoid haemorrhage (ICD9 852; ICD10 S06.6) was excluded. For the mortality analyses, a death was attributed to subarachnoid haemorrhage if the primary cause of death on certification was subarachnoid haemorrhage (ICD9 430 and ICD10 I60).

We pre-defined co-morbidities of interest which were: diabetes, cancer, respiratory disease, heart failure, peripheral arterial disease, atrial fibrillation, essential hypertension, renal failure, coronary heart disease and alcohol misuse. These co-morbidities were considered to be present if recorded as either a secondary diagnosis during the incident subarachnoid hospitalisation or as any diagnosis (primary or secondary) in the previous five years.

Specific interventional procedures amongst hospitalised patients were also identified using the relevant OPCS4 codes: neurosurgical clipping (L33.2), endovascular coiling (L35.1, L35.8) and CT angiography (L35.2). This data was only available from 1989 onwards. Postcode sectors of residence were used to assign socioeconomic status to all individuals based upon the Carstairs Morris index of deprivation[11].

Annual age and sex specific incidence rates of incident events for total, non-hospitalised fatal and hospitalised SAH were calculated using denominator data from the 1981, 1991 and 2001 censuses, with interpolation and extrapolation for the intra-census years. Rates were standardised to the 2001 Scottish census population using the direct standardisation method. Both hospitalised and total 30 days case fatality were estimated, the latter including non-hospitalised fatal incident events in the numerator and denominator. Poisson regression analysis was used to model the temporal trends in incidence of all first SAH with adjustment for age and socio-economic deprivation. Logistic regression analysis was used to model the temporal trends in 30 days hospitalised case fatality with adjustment for age, socio-economic deprivation and comorbidity. Fractional polynomial analysis was used to determine the best fitting curvi-linear relationship for the temporal trends of incidence and case fatality with study year. We determined *a priori *to conduct all analyses for men and women separately and to test for age by study year interactions. All analyses were carried out using STATA (Version 10, STATA Corp.) and a significance level of 0.05 was used throughout.

## Results

### Study populations

#### Hospitalised incident subarachnoid haemorrhage

Between 1^st ^January 1986 and 31^st ^December 2005, 10,113 patients were hospitalised in Scotland for incident SAH, 3,678 (36.4%) of which were men (see Table 1). Men had a mean age of 51.4 (SD 15.3) years and women 56.0 (15.5) years [p value for difference <0.001]. Comparing the final 5 years of the study period (2001 to 2005) with the initial 5 years (1986 to 1990), the mean age increased from 50.4 years (15.8) to 52.5 years (14.6) in men [p = 0.003] and from 55.6 years (15.4) to 57.1 years (15.3) in women [p = 0.01]. For both men and women, a socio-economic gradient was evident. Among men, 17.0% of cases occurred in the least deprived group compared with 21.2% in the most deprived group [p < 0.001]; the respective proportions were 18.2% and 21.7% in women [p < 0.001]. The most commonly coded co-morbid condition in men and women was hypertension (8.1% in men and 4.9% in women), followed by coronary heart disease (5.6% in men and 4.9% in women). Intervention by endovascular coiling or neurosurgical clipping was carried out in 34.9% of men and 44.8% of women hospitalised in 2005. During the period 1989 to 2005, of those patients receiving neurosurgical clipping, 49.1% were aged under 50 years, and of those receiving neurosurgical coiling, 46.0% were under 50. The proportion of patients undergoing intervention is in line with those observed in the literature[3,12,13].

**Table 1:** Descriptive statistics for men and women hospitalised with an incident SAH between 1^st ^January 1986 and 31^st ^December 2005

	Men	Women
**Overall**	3,678 (36.4%)	6,435 (63.6%)
**Age (years) - mean (SD)**	51.4 (15.3)	56.0 (15.5)
**Age (years) by study year - mean (SD)**		
1986 to 1990	50.4 (15.8)	55.6 (15.4)
1991 to 1995	50.3 (15.4)	54.5 (15.6)
1996 to 2000	52.1 (15.3)	56.8 (15.6)
2001 to 2005	52.5 (14.6)	57.1 (15.3)
**Age groups (years)**		
< 40	823 (22.4%)	974 (15.1%)
40-49	830 (22.6%)	1247 (19.4%)
50-59	880 (23.9%)	1493 (23.2%)
60-69	675 (18.4%)	1363 (21.2%)
70+	470 (12.8%)	1358 (21.1%)
**Socio-economic deprivation**		
1-least deprived	603 (17.0%)	1134 (18.2%)
2	705 (19.9%)	1221 (19.6%)
3	714 (20.2%)	1205 (19.3%)
4	766 (21.6%)	1321 (21.2%)
5- most deprived	752 (21.2%)	1352 (21.7%)
Missing	138 (3.8%)	202 (3.1%)
**Comorbid diagnoses**		
Alcohol misuse	165 (4.5%)	111 (1.7%)
Atrial fibrillation	80 (2.2%)	90 (1.4%)
Cancer	86 (2.3%)	167 (2.6%)
Coronary heart dis.	206 (5.6%)	316 (4.9%)
Diabetes	86 (2.3%)	105 (1.6%)
Hypertension	299 (8.1%)	583 (9.1%)
Heart failure	59 (1.6%)	84 (1.3%)
Peripheral arter. dis.	130 (3.5%)	195 (3.0%)
Renal failure	36 (1.0%)	57 (0.9%)
Respiratory disease	111 (3.0%)	247 (3.8%)
Any condition above	844 (22.9%)	1416 (22.0%)

#### Non-hospitalised fatal incident subarachnoid haemorrhage

Between 1^st ^January 1986 and 31^st ^December 2005, there were 1,943 fatal incident SAH events in Scotland, in which no hospitalisation occurred. 741 (38.1%) of cases were men. The mean age of men (54.8 years [SD 15.1]) was less than that of women (60.4 years [15.1]; (95% CI -5.3 to -4.0, p < 0.001). For both men and women, those whose incident event was fatal were older than those whose incident event led to hospital admission [p < 0.001]. Comparing the final 5 years of the study period (2001 to 2005) with the initial 5 years (1986 to 1990), the mean age increased from 52.7 years (SD 14.6) to 57.2 years (SD 15.1) in men [p = 0.004] and minimally from 60.4 years (SD 14.8) to 60.8 years (SD 15.2) in women [p = 0.9]. As for hospitalised incident SAH there was a socioeconomic gradient; 18.4% of affected men were in the least deprived category compared with 22.9% in the most deprived category [p = 0.03]; the respective proportions in women were 16.1% and 22.9% [p < 0.001].

### Age standardised population rates

**Figure 1:**
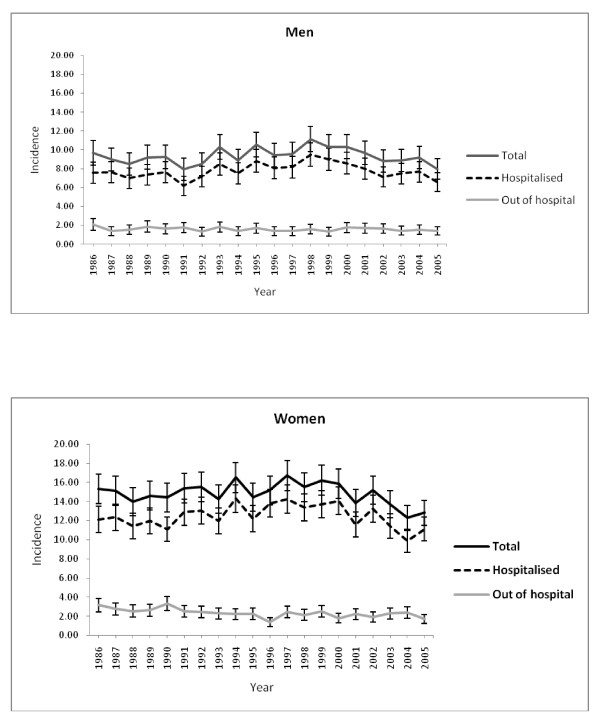
**Observed age standardised rates (per 100,000) for hospitalised, out of hospital deaths and total SAH in men and women**.

Overall age standardised rates for incident SAH (per 100,000 population) were greater in women than in men (Figure 1). In men and women rates were relatively stable across the 20 year period [men, 9.7 (8.4 to 8.7) in 1986 and 7.9 (6.9 to 9.1) in 2005; women, 15.3 (13.8 to 16.8) in 1986 and 12.8 (11.5 to 14.2) in 2005]. After adjustment, the relative risk of incident subarachnoid haemorrhage in 2005 compared to 1986 was 0.99 (0.88 to 1.10), p = 0.8 in men and 0.95 (0.87 to 1.03), p = 0.2 in women.

### 30 day case fatality

Overall (hospitalised and non-hospitalised) 30 day case fatality increased substantially with age in both men and women, increasing from 28.5% in men and 24.6% in women aged <40 years to 59.7% in men and 57.3% in women in those aged 70 and over (Table 2). In hospitalised patients only, 30 day case fatality demonstrated a similar relationship with age. In general, both overall and hospitalised case fatality decreased over the course of the study period. For both men and women the largest reductions in case fatality were observed for those aged between 40-59 years. In hospitalised men aged 40 to 49 years, 30 day case fatality fell during the study period, from 30% in the period 1986-1990 to 21.4% in the period 2001-2005 (32.6% and 18.8% in women respectively). There were no improvements in case fatality for patients aged 70 years and older.

**Table 2:** Hospitalised and overall 30 days case fatality by age, sex and study year

Hospitalised 30 days case fatality (%)							
		**Age groups (years)**					
	**Study year**	** < 40**	**40-49**	**50-59**	**60-69**	**70+**	**All ages**
**Men**	**1986 to 1990**	21.8	30.0	27.4	33.3	46.2	30.0
	**1991 to 1995**	15.5	22.4	23.7	32.3	46.7	25.7
	**1996 to 2000**	17.3	21.3	26.3	39.2	52.1	29.3
	**2001 to 2005**	17.7	21.4	17.6	27.5	49.6	24.5
	**All years**	18.1	23.6	23.8	33.2	48.9	27.4
							
**Women**	**1986 to 1990**	18.1	32.6	31.0	38.2	46.0	33.9
	**1991 to 1995**	17.7	26.2	26.1	32.2	40.9	28.5
	**1996 to 2000**	13.6	22.3	25.8	30.4	48.2	29.7
	**2001 to 2005**	17.4	18.8	24.4	32.6	47.1	29.1
	**All years**	16.7	24.7	26.7	33.3	45.9	30.2
							

**Overall 30 days case fatality (%)**							

		**Age groups (years)**					
	**Study year**	** < 40**	**40-49**	**50-59**	**60-69**	**70+**	**All ages**
**Men**	**1986 to 1990**	33.1	41.9	43.7	48.1	55.5	42.9
	**1991 to 1995**	27.1	32.2	38.8	45.2	59.3	38.4
	**1996 to 2000**	26.3	32.8	37.0	48.7	60.9	39.6
	**2001 to 2005**	27.0	32.8	33.2	40.6	62.3	37.6
	**All years**	28.5	34.6	38.0	45.6	59.7	39.6
							
**Women**	**1986 to 1990**	27.9	43.0	45.2	50.0	60.7	46.8
	**1991 to 1995**	25.4	37.1	37.7	42.6	52.9	39.5
	**1996 to 2000**	19.3	31.8	33.8	40.4	57.2	38.8
	**2001 to 2005**	25.8	27.5	37.6	42.7	58.0	40.2
	**All years**	24.6	34.7	38.4	44.0	57.3	41.2

In the adjusted analyses, there was a significant interaction between age and study year when modelling hospitalised 30 day case fatality with a similar size of interaction effect for men and women (interaction p value for all patients = 0.001]. In those aged less than 70 years the odds ratio (OR) of odds of death at 30 days in 2005 compared to odds of death at 30 days in 1986 was 0.64 (0.54 to 0.76), p < 0.001. In those aged 70 years and above the corresponding OR was 1.14 (0.83 to 1.56), p = 0.4.

## Discussion

### Overall Incidence of SAH in Men and Women

Subarachnoid haemorrhage accounts for nearly one third of all stroke related years of potential life lost before age 65[3]. Between 1986 and 2005, 12,056 individuals in Scotland experienced an incident SAH. Of these 84% survived to reach hospital. Overall age standardised incidence rates were greater in women [14.8 (13.4 to 16.3)] than in men [9.4 (8.1 to 10.6)] throughout the study period, with an overall female to male ratio of 1.59. A recent systematic review reported an overall incidence of 11.5 per 100,000 person-years in women and 9.2 in men with an overall female to male ratio of 1.24[14]. The reasons for the frequently reported higher incidence of SAH in women compared to men is unknown, although hormonal factors have been postulated[15]. Although the evidence is conflicting, some studies have suggested that hormone replacement therapy is associated with a reduced risk of SAH[4,14].

### Temporal Trends in Incidence of SAH

Age-adjusted incidence of SAH in both men and women was relatively stable across the study period. Other studies[8,9,16] observed a similar pattern, although comparison is hindered by the use of different time periods.

The absence of a decline in the incidence of SAH in Scotland, despite intensive efforts to reduce the prevalence of risk factors[17] such as smoking, hypertension and alcohol misuse is surprising and at odds with the declining trend seen in the incidence of ischaemic stroke in Scotland[18]. It may reflect the younger age profile of SAH compared with ischaemic stroke and a lack of impact of risk factor reduction measures in younger individuals. It may also be that the importance of genetic or other unrecognised risk factors in SAH are higher than in ischaemic stroke, meaning that any health education and behavioural change programmes will have less impact on this type of stroke.

### 30 Day Case Fatality following Incident SAH

Early case fatality following SAH is high. In our study 16.1% of individuals (16.8% of men and 15.7% of women) with incident SAH died out of hospital. This is identical to the rate of out of hospital case-fatality reported in a study that examined 800 incident cases in which the diagnosis was verified by either CT or necropsy in 99%[13], and consistent with a meta-analysis which reported a probability of sudden death ranging from 3% to 21%[19].

30 day case fatality was also high at 24.5% in men and 29.1% in women even in the period 2001 to 2005. These figures are similar to those reported from other countries. In the Swedish Hospital Discharge and Cause of Death Registry, 30.5% of men and 32.5% of women died within 28 days between 1987 and 2002[7]. Case fatality increased steeply with age, rising from 27.0% in men aged less than 40 years to 62.3% in those aged 70 and over during the period 2001-2005. Again these findings are consistent with Swedish studies which also observed increasing case fatality with increasing age[7,20]. It has been suggested that this may be due, in part, to less aggressive management of SAH in the elderly[7] although there is little evidence to support this.

### Temporal Trends in 30 Day Case Fatality following Incident SAH

The most important new finding of our study is the substantial decline in 30 day case fatality rates in men and women over the study period. These declines were greatest in those aged 40 to 59 years. For the 40 to 49 year age group, case fatality fell from 30.0% in 1986-1990 to 21.4% in 2001 to 2005 in men and from 32.6% to 18.8% in women, and was not changed substantially by adjustment for age, socio-economic deprivation and co-morbidity. Overall case fatality, including out of hospital deaths, also declined, although the relative magnitude of the decline was less than for hospitalised case fatality. There are few recently published data with which to compare these findings and none of the published reports have examined age specific trends in case fatality in men and women. The Swedish Hospital Discharge and Cause of Death Registry Study reported an absolute risk reduction in 28 day case fatality of 5.8% in patients under 70 years and 3.5% in patients of 70 years and older comparing the period 1995-2002 and 1987-1994[7]. A number of other studies have reported stable case fatality rates for SAH. These include the Dijon Stroke Study, the Perth Community Stroke Study and a study carried out using the United States National Hospital Discharge Survey[21]. However, the Dijon and Perth Studies were based on small numbers[8,9]. The National Hospital Discharge Survey Study examined in-hospital mortality rates for SAH hospitalisations between 1986 and 2001[21]. During this time period the number of hospitalisations increased along with the mean age of those hospitalised. The analyses of in-hospital case fatality were crude and did not take into account the changing case mix over time. This may have masked any improvement in outcome which varied by age or sex. In an update of a previous meta-analysis, including only population-based prospective studies, Nieuwkamp et al.[10] examined changes in case fatality in the period 1972 to 2003. 30 day case fatality did not appear to have declined after the results were adjusted for age and sex. However there was considerable heterogeneity in case finding and diagnostic approaches among studies combined.

Changes in the investigation, medical and interventional management of SAH, including the introduction of CT angiography, use of nimodipine[22] and endovascular coiling[6], may have contributed to the reduction observed in 30 day case fatality. An audit[23] of the management of and referral patterns for SAH patients conducted in the South Thames area in 1997, showed that for many patients care was deficient. In particular, delays in obtaining CT scans, and the consequent delays in diagnoses, prevented the early commencement of treatment and appropriate referrals. Moves towards defined patient pathways, increased understanding of the need for rapid action and improved adherence to guidelines may also have resulted in reductions in case fatality over recent years.

### Strengths and weaknesses of the study

This study has a number of strengths. As a result of individual patient record linkage we were able to identify incident cases of both fatal out of hospital and hospitalised SAH in an entire population over nearly 3 decades. There are some limitations to our study. We used routinely collected and coded hospital discharge data. These are audited by ISD on a four yearly cycle and SAH is coded in a principal diagnostic position at discharge with a high degree of accuracy, 95%[24]. Our definition of a first SAH used a time dependant look back period. It is possible that a very small number of individuals may have been hospitalised previously for SAH outside this period. Our study may have excluded a small number of individuals with mild symptoms who were not hospitalised.

## Conclusions

30 day case SAH fatality rates have declined substantially, especially in middle age patients. It is important to ensure that the observed higher risk in the elderly is not due to under-diagnosis or under-treatment.

Despite improvements in cardiovascular risk factors at a population level, the incidence of SAH has not declined. Subarachnoid haemorrhage remains an important public health issue, especially in women.

## Competing interests

The authors declare that they have no competing interests.

## Authors' contributions

The study was conceived by K MacIntyre and MG. K Macpherson and JL undertook the data analysis. The manuscript was prepared by K Macpherson with assistance from JL and K MacIntyre. PJ, MG, JC, AR, AB, MW, PL, SC and JM provided input to the design of the study, assisted with the interpretation of the analyses and undertook review of the study manuscript.

All authors had full access to all of the data (including statistical reports and tables) in the study and can take responsibility for the integrity of the data and the accuracy of the data analysis. All authors read and approved the final draft.

## Pre-publication history

The pre-publication history for this paper can be accessed here:

http://www.biomedcentral.com/1471-2377/11/38/prepub
